# Supplementary material for: Jatrolignans C and D: New Neolignan Epimers from Jatropha curcas
Source: Molecules. 2022 May 31;27(11):3540. doi: 10.3390/molecules27113540 (PMC9182123; doi:10.3390/molecules27113540)
Supplement: Supplementary file 1 [file molecules-27-03540-s001.zip › molecules-1745083-supplementary.pdf]

Supporting Information for Jatrolignans C and D: New neolignan epimers from *Jatropha curcas*

Yi-Lin He[1,2], Pei-Zhi Huang [1], Hong-Ying Yang [1], Wei-Jiao Feng [1], Zhao-Cai Li[3] and Kun Gao[1,*]

[1] State Key Laboratory of Applied Organic Chemistry, College of Chemistry and Chemical Engineering, Lanzhou University, Lanzhou 730000, People's Republic of China

[2] Research Institute, Lanzhou Jiaotong University, Lanzhou 730070, People's Republic of China

[3] State Key Laboratory of Veterinary Etiological Biology, Lanzhou Veterinary Research Institute, Chinese Academy of Agricultural Sciences, Lanzhou, 730046, China

* Correspondence: npchem@lzu.edu.cn (K. Gao)

List of Figures.

Figure S1. ^{1}H NMR spectrum of compound **1** (600 MHz, MeOD) 1
Figure S2. ^{13}C NMR spectrum of compound **1** (150 MHz, MeOD) 1
Figure S3. HSQC spectrum of compound **1** (150 MHz, MeOD) 2
Figure S4. HMBC spectrum of compound **1** (150 MHz, MeOD) 2
Figure S5. ^{1}H-^{1}H COSY spectrum of compound **1** (600 MHz, MeOD) 3
Figure S6. NOESY spectrum of compound **1** (600 MHz, MeOD) 3
Figure S7. HR-ESIMS of compound **1** 4
Figure S8. IR spectrum of compound **1** 4
Figure S9. ^{1}H NMR spectrum of compound **2** (600 MHz, MeOD) 5
Figure S10. ^{13}C NMR spectrum of compound **2** (150 MHz, MeOD) 5
Figure S11. HSQC spectrum of compound **2** (150 MHz, MeOD) 6
Figure S12. HMBC spectrum of compound **2** (150 MHz, MeOD) 6
Figure S13. ^{1}H-^{1}H COSY spectrum of compound **2**(600 MHz, MeOD) 7
Figure S14. NOESY spectrum of compound **2** (600 MHz, MeOD) 7
Figure S15. HR-ESIMS of compound **2** 8
Figure S16. IR spectrum of compound **2** 8
Table S1 Gibbs free energies and Boltzmann populations of Compound **1** 9
Table S2 Gibbs free energies and Boltzmann populations of Compound **2** 9
Figure S17. The experimental and calculated UV spectrum of compound **1** 10
Figure S18. The experimental and calculated UV spectrum of compound **2** 10
Figure S19. HPLC spectra of the MeOH extract and compound **1** and **2.** 11
Table S3. ECD-Measurement Information 11
Computational Details 12

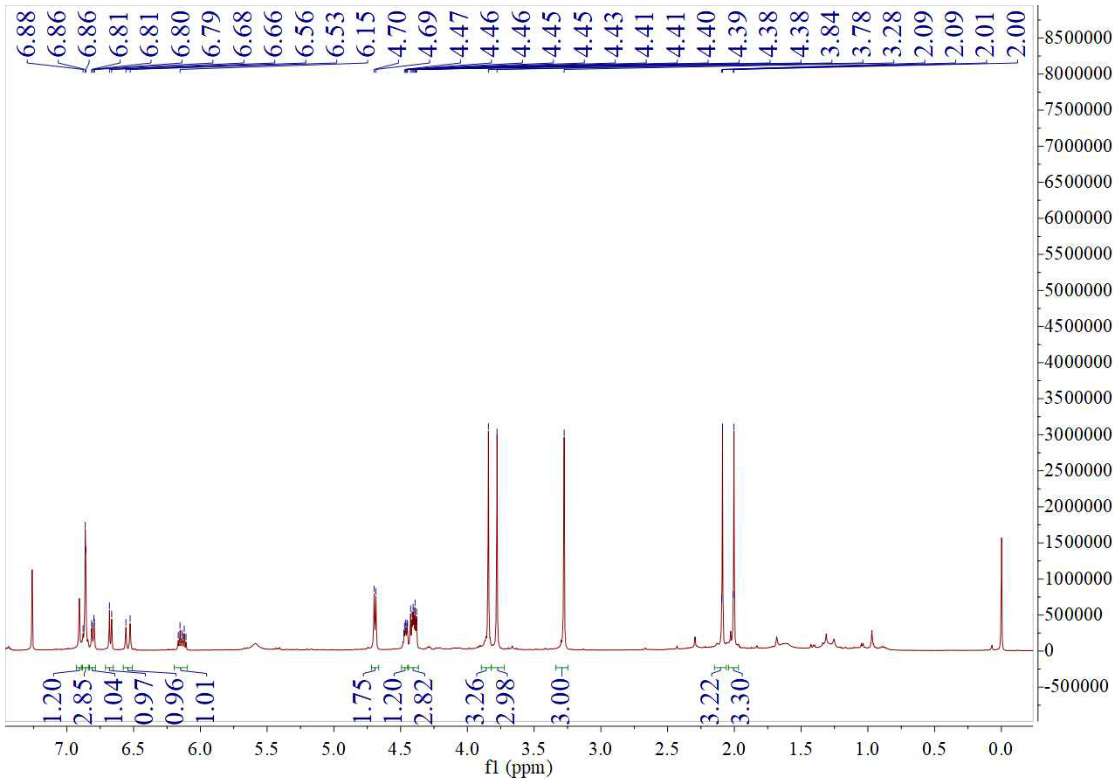

Figure S1. ^{1}H NMR spectrum of compound **1** (600 MHz, MeOD).

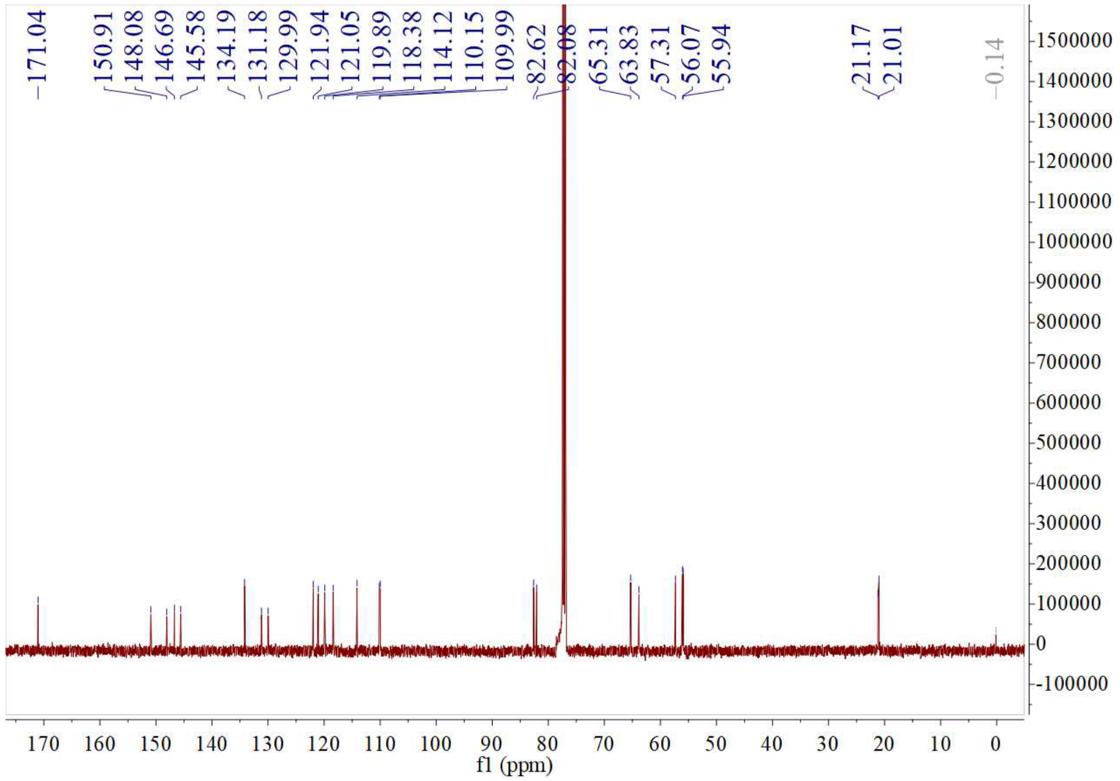

Figure S2. ^{13}C NMR spectrum of compound **1** (150 MHz, MeOD).

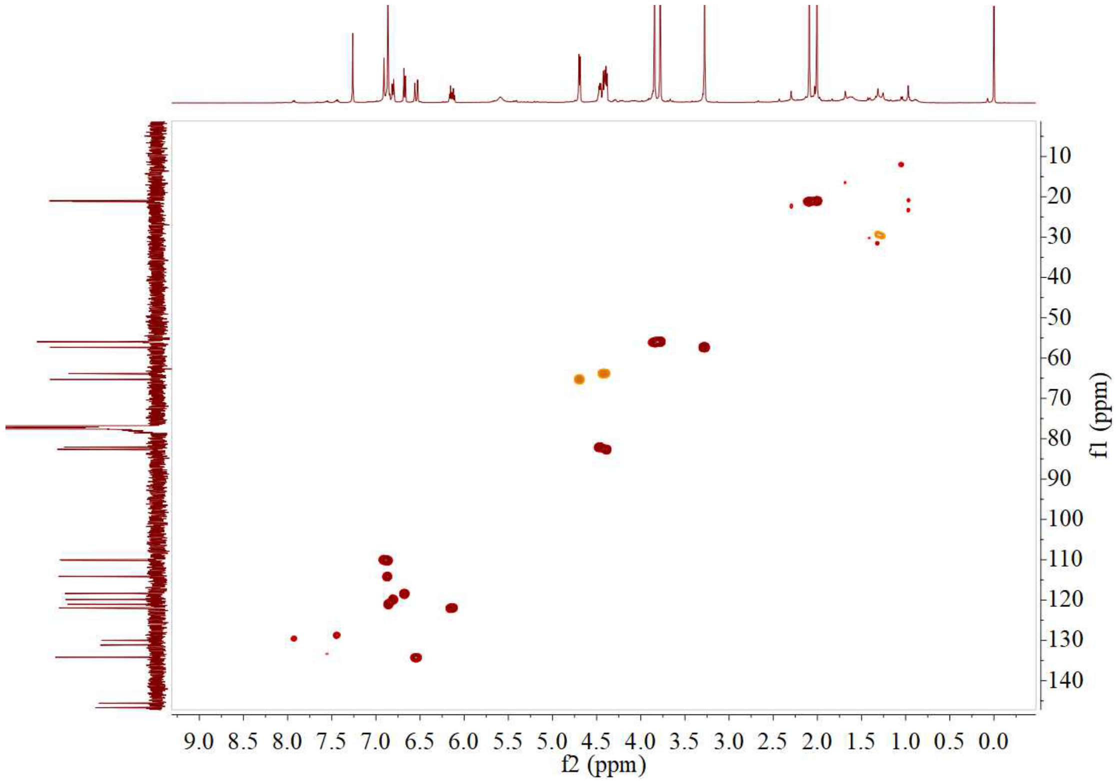

Figure S3. HSQC spectrum of compound **1** (150 MHz, MeOD).

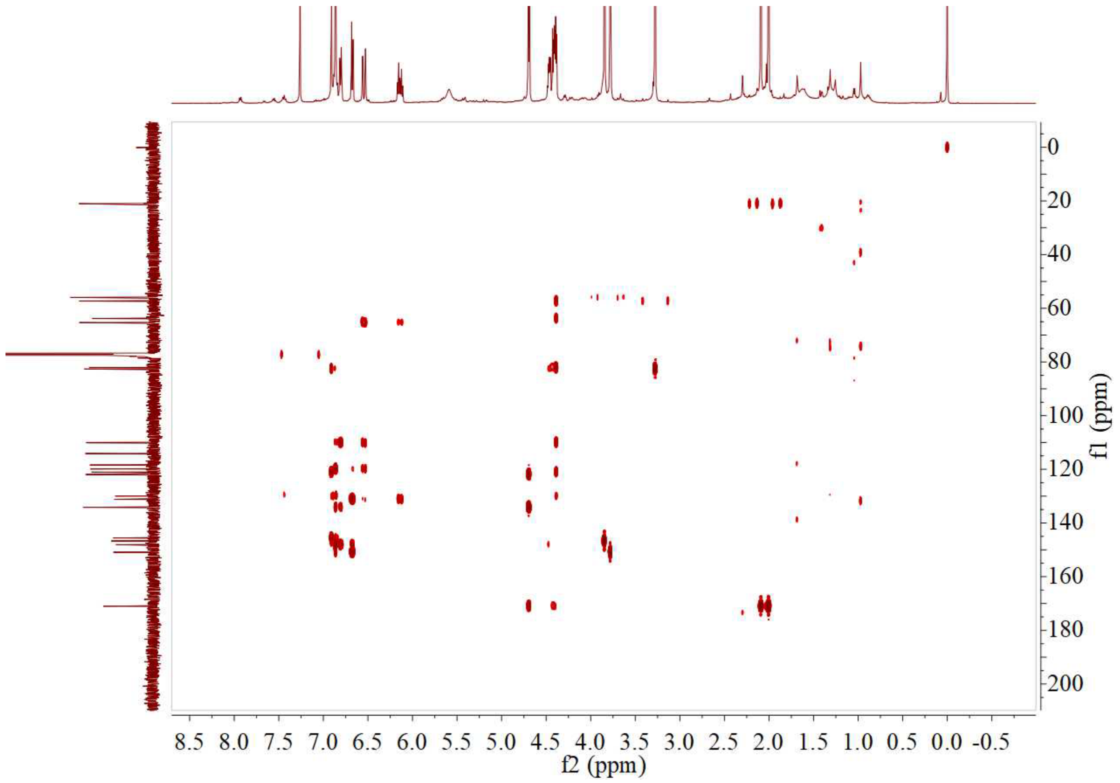

Figure S4. HMBC spectrum of compound **1** (150 MHz, MeOD).

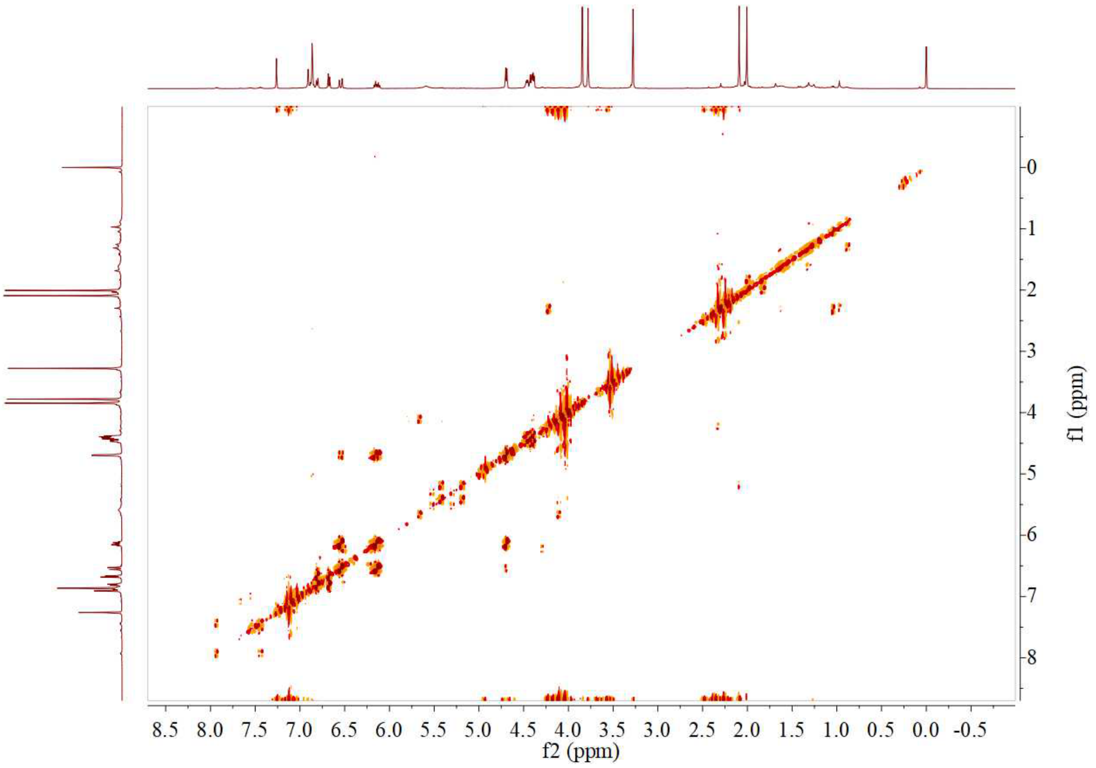

Figure S5. ^{1}H-^{1}H COSY spectrum of compound **1** (600 MHz, MeOD).

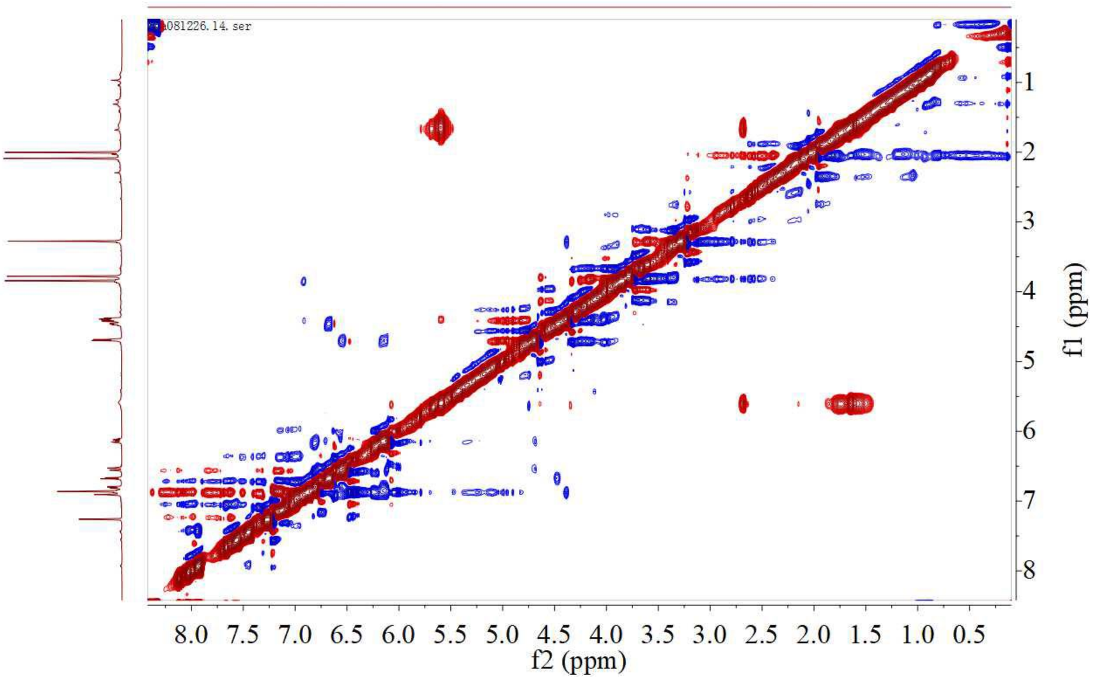

Figure S6. NOESY spectrum of compound **1** (600 MHz, MeOD).

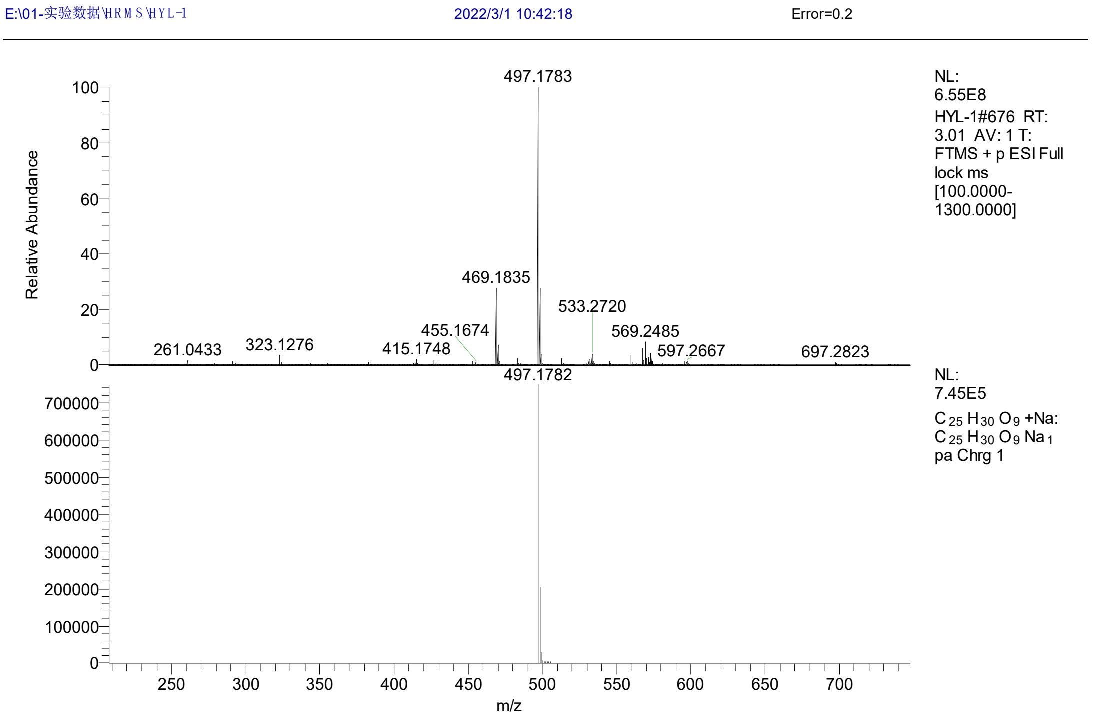

Figure S7. HR-ESIMS of compound **1.**

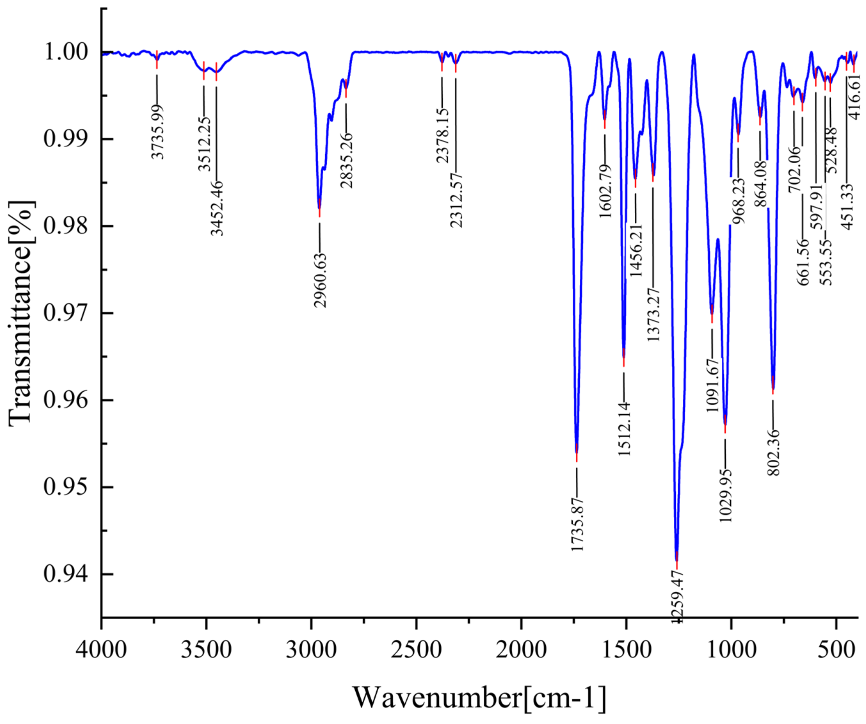

Figure S8. IR spectrum of compound **1.**

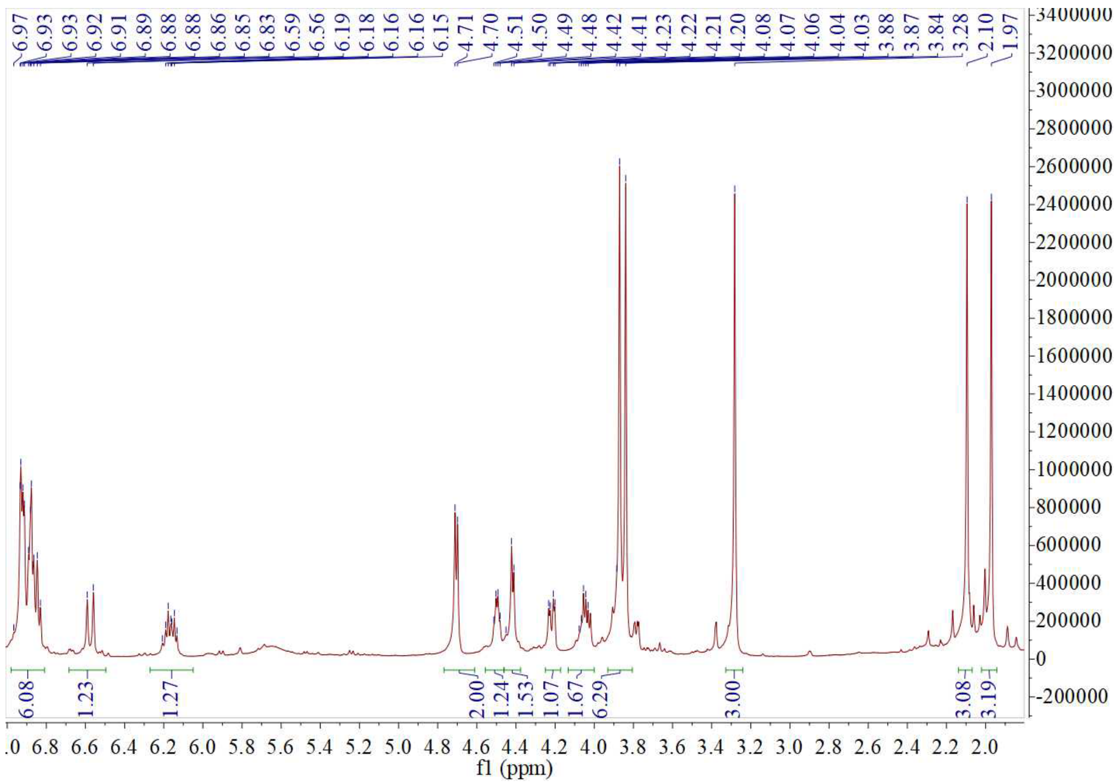

Figure S9. ^{1}H NMR spectrum of compound **2** (600 MHz, MeOD).

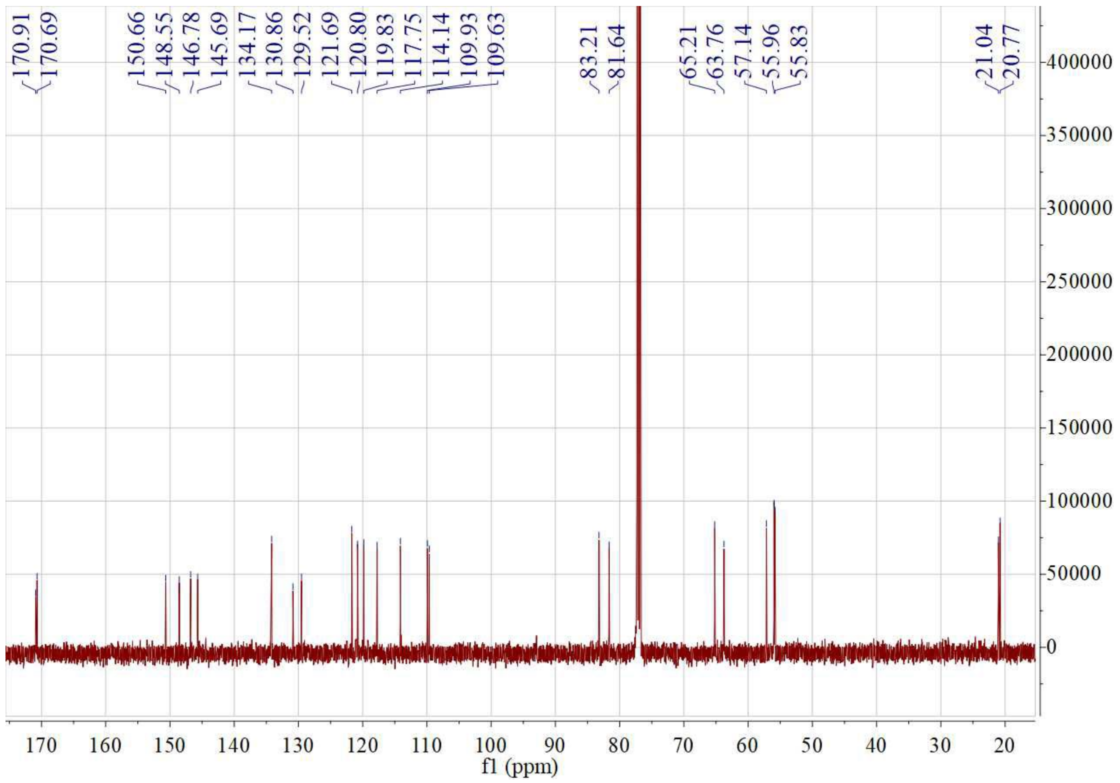

Figure S10. ^{13}C NMR spectrum of compound **2** (150 MHz, MeOD).

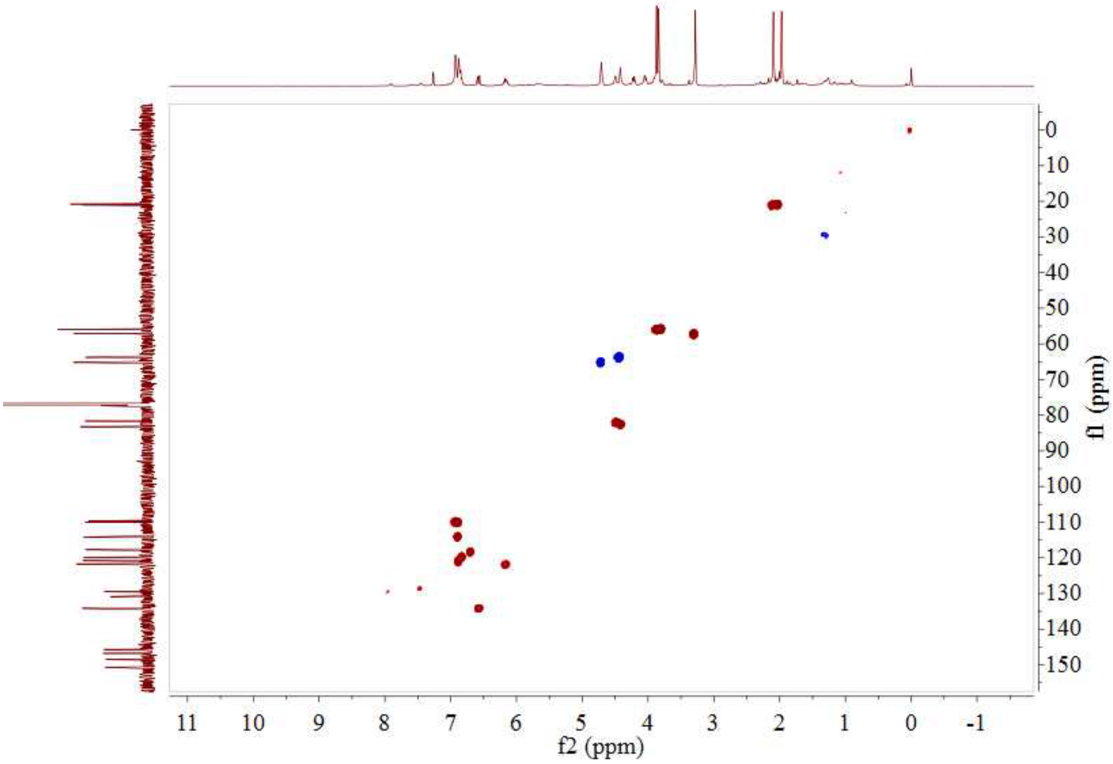

Figure S11. HSQC spectrum of compound **2** (150 MHz, MeOD).

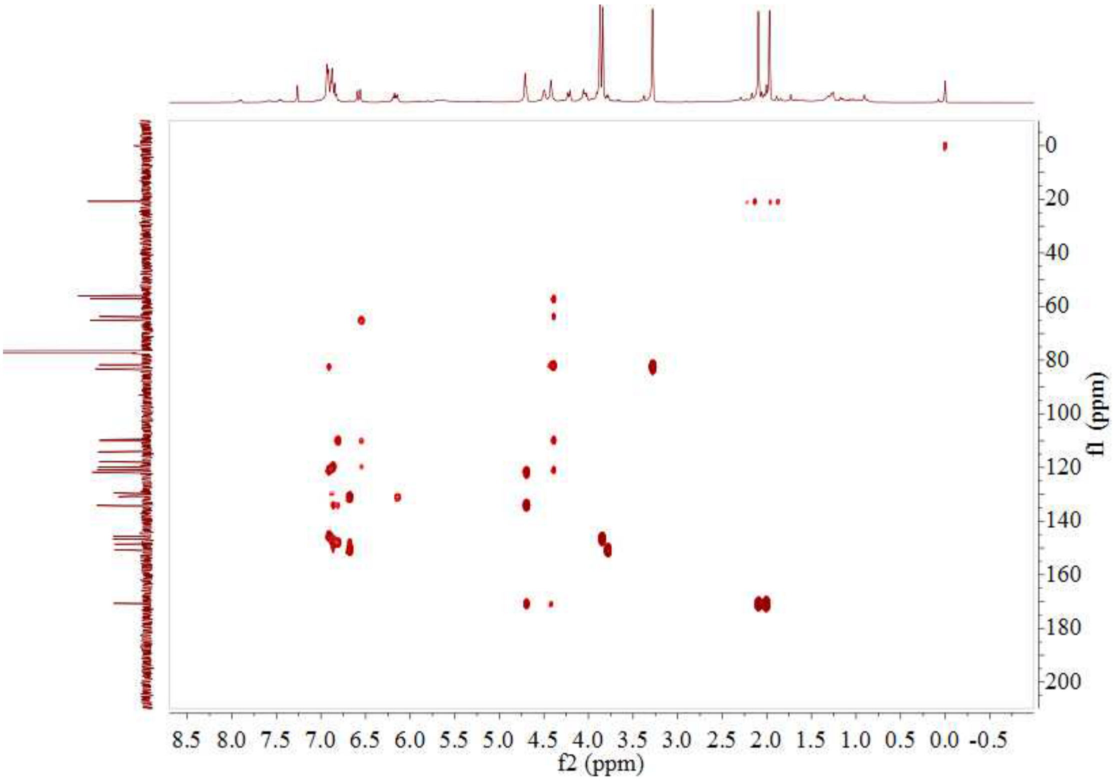

Figure S12. HMBC spectrum of compound **2** (150 MHz, MeOD).

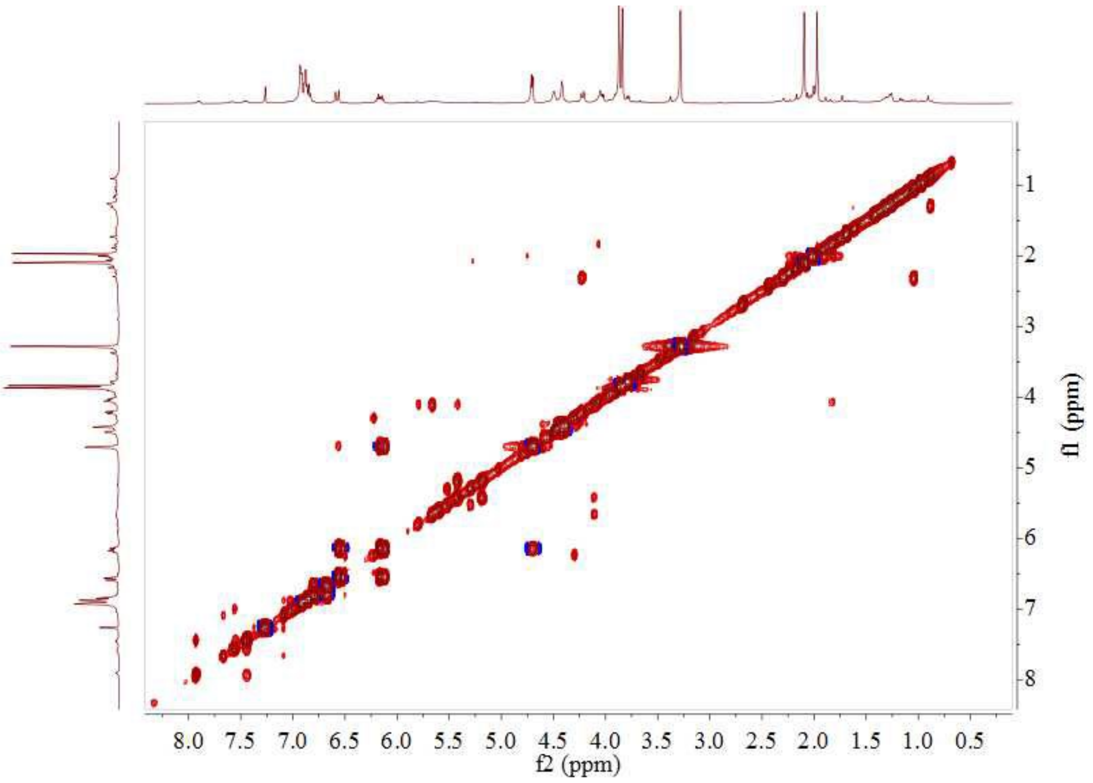

Figure S13. ^{1}H-^{1}H COSY spectrum of compound **2**(600 MHz, MeOD).

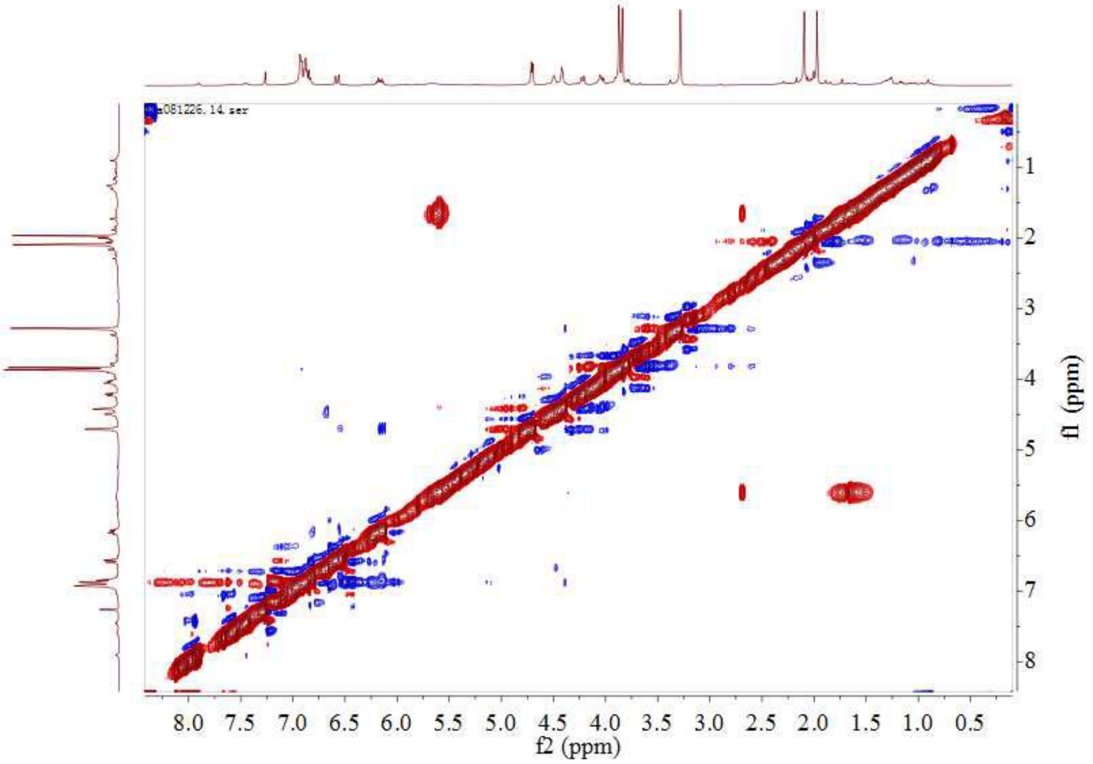

Figure S14. NOESY spectrum of compound **2** (600 MHz, MeOD).

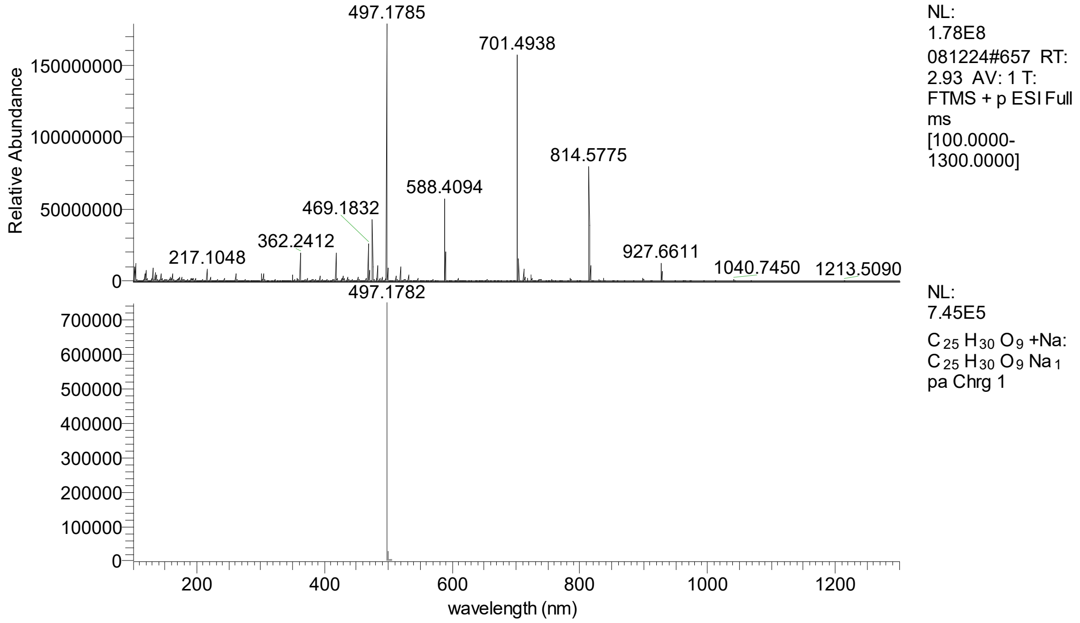

Figure S15. HR-ESIMS of compound **2.**

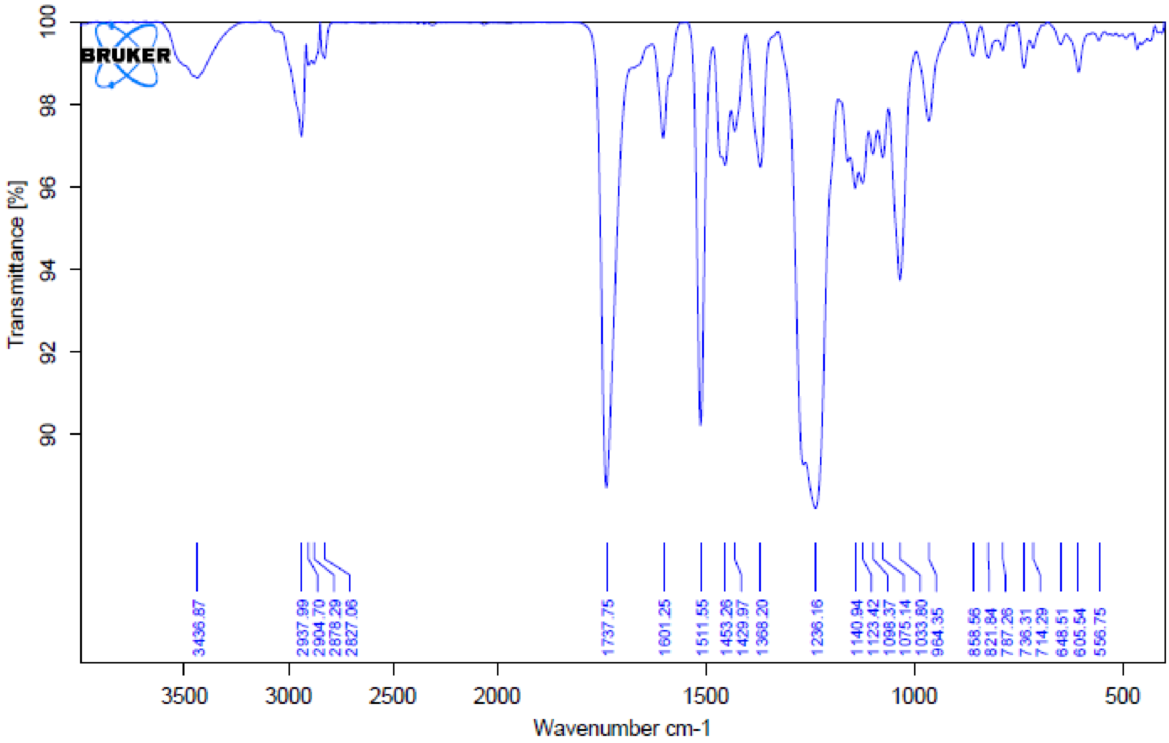

Figure S16. IR spectrum of compound **2.**

Table S1. Gibbs free energies and Boltzmann populations of Compound **1.**

Conformers	ΔG (kcal/mol)	P (%)
Conf 1	0.00	42.26
Conf 2	0.29	25.96
Conf 3	0.38	22.08
Conf 4	1.12	6.41
Conf 5	1.93	1.62
Conf 6	2.17	1.09
Conf 7	2.94	0.29
Conf 8	3.23	0.18
Conf 9	4.01	0.05
Conf 10	4.25	0.03
Conf 11	4.68	0.02
Conf 12	5.73	0.00

Table S2. Gibbs free energies and Boltzmann populations of Compound **2.**

Conformers	ΔG (kcal/mol)	P (%)
Conf 1	0.00	79.58
Conf 2	1.17	11.03
Conf 3	1.33	8.48
Conf 4	3.11	0.42
Conf 5	3.48	0.22
Conf 6	3.96	0.10
Conf 7	4.21	0.07
Conf 8	4.27	0.06
Conf 9	4.51	0.04
Conf 10	5.55	0.01
Conf 11	6.09	0.00
Conf 12	6.15	0.00

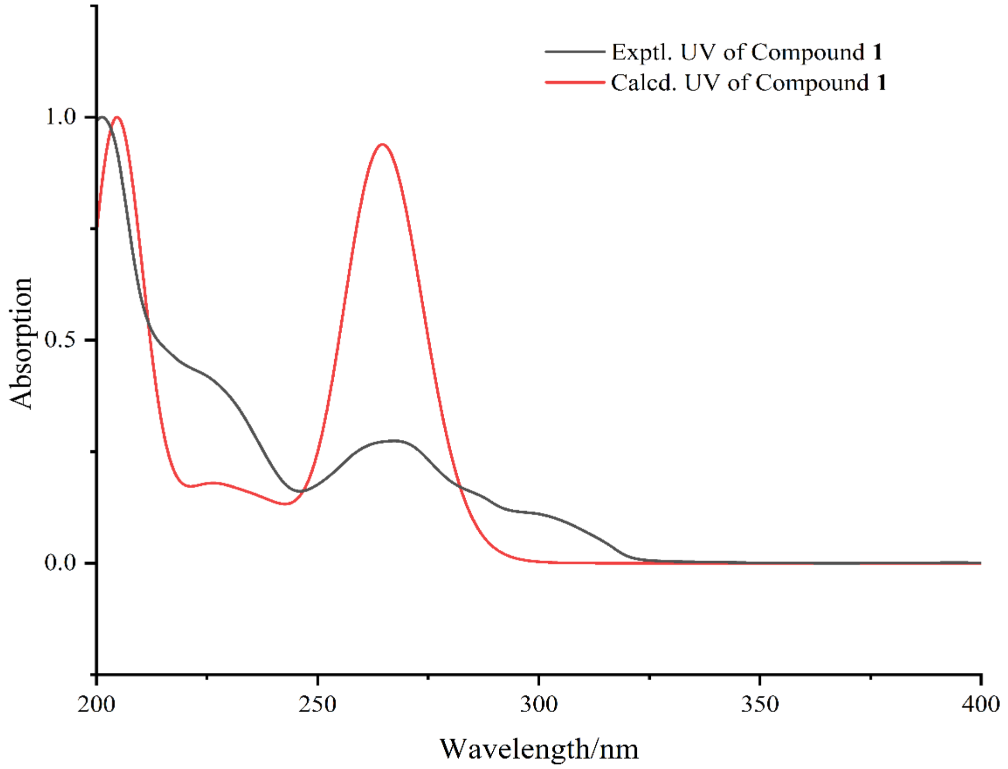

Figure S17. The experimental and calculated UV spectrum of compound **1.**

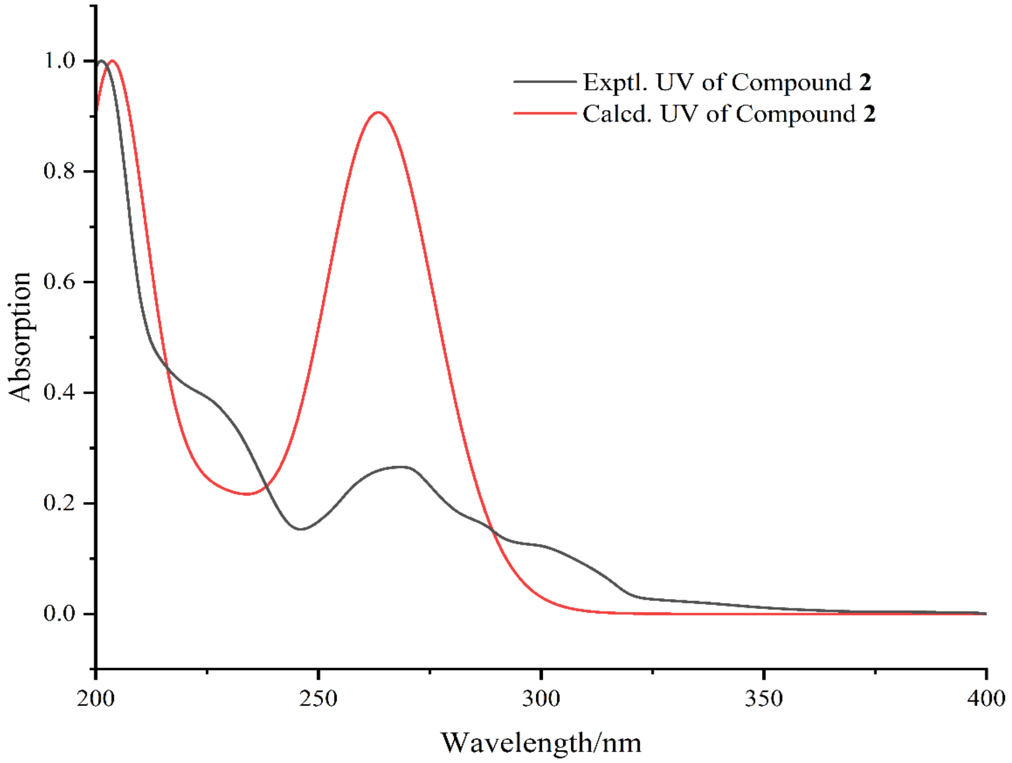

Figure S18. The experimental and calculated UV spectrum of compound **2.**

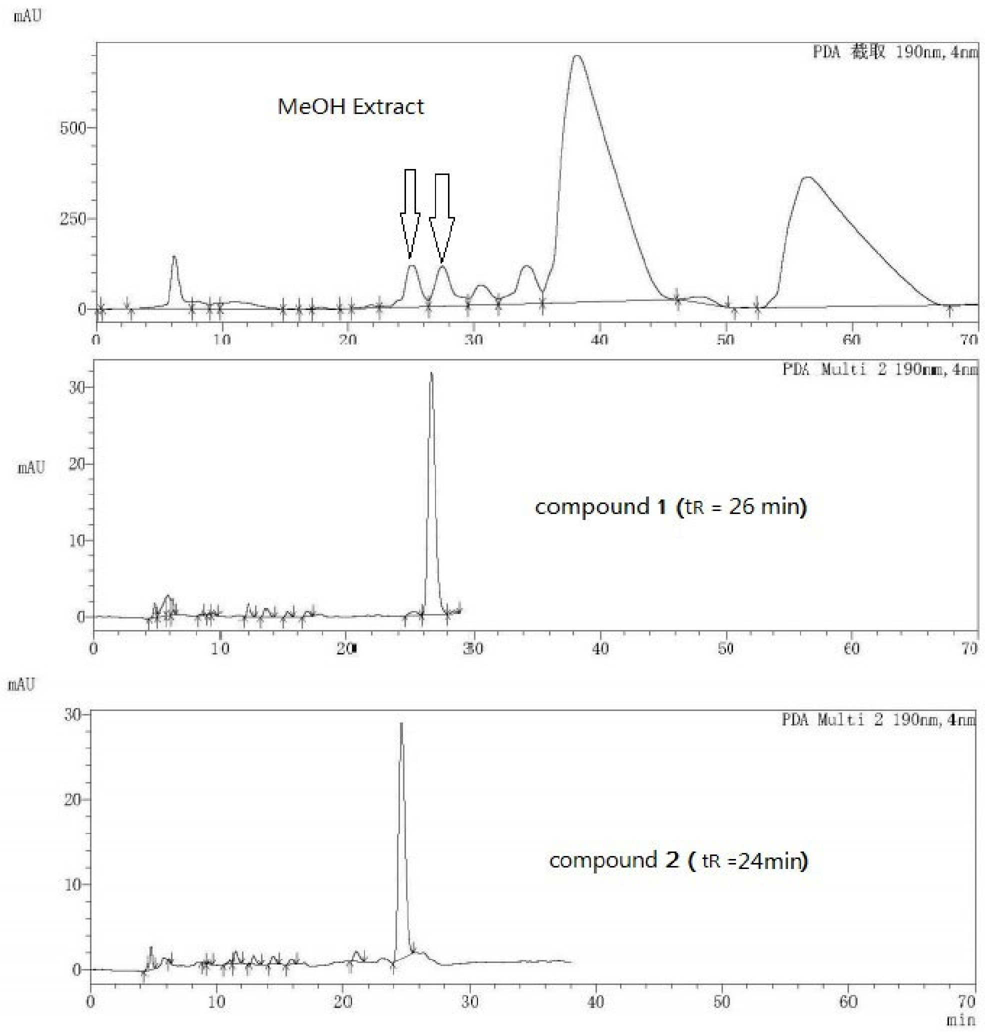

Figure S19. HPLC spectra of the MeOH extract and compounds **1** and **2.**

Table S3. ECD-Measurement Information.

Instrument name	J-1500	CD Overload detect	192
Model name	J-1500	Photometric mode	CD
Serial No.	D062061638	Measure range	400 - 200 nm
		Data pitch	1 nm
Detector	PM-539	CD scale	200 mdeg/0.1 dOD
Detector S/N	D062061638	FL scale	200 mdeg/0.1 dOD
Lock-in amp.	X mode	D.I.T.	0.5 sec
HT volt	Auto	Bandwidth	1.00 nm
		Start mode	Immediately
Accessory	PTC-517	Scanning mode	Continuous
Accessory S/N	C032961646	Scanning speed	200 nm/min
Temperature	4.00 C	Baseline correction	Baseline
Control sensor	Holder	Shutter control	Auto
Monitor sensor	Holder	Accumulations	1
Measurement date	2021.10.21	N2 Flowmeter	Manual

Computational Details

The theoretical calculation assay was determined by TD-DFT method. The initial conformer ensemble was constructed by xtb program [22] with molecular metadynamic [23] method and conducted preliminary geometry optimization by crest [24] with GFN2-xtb [25-27] method. The conformers optimized previously then performed a middle-level geometry optimization by ORCA [28,29] with B97-3c [30] method, all the screening and sorting procedures of conformers were performed by molclus program [31]. Conformers with energy within 7 kcal/mol energy window from the conformation ensemble were chosen to proceed further frequency calculation and single point energy calculation, to obtain the free energy correction of each conformer by Gaussian16 [32], frequency calculation was performed with B3LYP-D3(BJ)/def2-SVP method in gaseous environment. High accurate single point energy of all optimized conformers calculated with PWPB95-D4/def2-QZVPP method, the calculation of Gibbs free energy and Boltzmann population was conducted by molclus program. The ECD spectra was calculated by TD-DFT with M062X/def2-TZVP(-f) method and fitted the lowest 10 excited states with Gaussian function. All ECD and single point energy calculations were calculated by ORCA 4.2.1 using SMD [33] solvation model and Methanol was specified as the solvent which was consistent with experiment condition, The ECD spectra with Boltzmann population weighting was processed with SpecDis [34].
